# *Bunyaviridae* RNA Polymerases (L-Protein) Have an N-Terminal, Influenza-Like Endonuclease Domain, Essential for Viral Cap-Dependent Transcription

**DOI:** 10.1371/journal.ppat.1001101

**Published:** 2010-09-16

**Authors:** Juan Reguera, Friedemann Weber, Stephen Cusack

**Affiliations:** 1 European Molecular Biology Laboratory, Grenoble Outstation, Grenoble, France; 2 Unit of Virus Host-Cell Interactions (UMI 3265), UJF-EMBL-CNRS, Grenoble, France; 3 Department of Virology, Institute for Medical Microbiology and Hygiene, Freiburg, Germany; 4 Institute for Virology, Philipps University Marburg, Marburg, Germany; Institut Pasteur, France

## Abstract

Bunyaviruses are a large family of segmented RNA viruses which, like influenza virus, use a cap-snatching mechanism for transcription whereby short capped primers derived by endonucleolytic cleavage of host mRNAs are used by the viral RNA-dependent RNA polymerase (L-protein) to transcribe viral mRNAs. It was recently shown that the cap-snatching endonuclease of influenza virus resides in a discrete N-terminal domain of the PA polymerase subunit. Here we structurally and functionally characterize a similar endonuclease in La Crosse orthobunyavirus (LACV) L-protein. We expressed N-terminal fragments of the LACV L-protein and found that residues 1-180 have metal binding and divalent cation dependent nuclease activity analogous to that of influenza virus endonuclease. The 2.2 Å resolution X-ray crystal structure of the domain confirms that LACV and influenza endonucleases have similar overall folds and identical two metal binding active sites. The *in vitro* activity of the LACV endonuclease could be abolished by point mutations in the active site or by binding 2,4-dioxo-4-phenylbutanoic acid (DPBA), a known influenza virus endonuclease inhibitor. A crystal structure with bound DPBA shows the inhibitor chelating two active site manganese ions. The essential role of this endonuclease in cap-dependent transcription was demonstrated by the loss of transcriptional activity in a RNP reconstitution system in cells upon making the same point mutations in the context of the full-length LACV L-protein. Using structure based sequence alignments we show that a similar endonuclease almost certainly exists at the N-terminus of L-proteins or PA polymerase subunits of essentially all known negative strand and cap-snatching segmented RNA viruses including arenaviruses (2 segments), bunyaviruses (3 segments), tenuiviruses (4–6 segments), and orthomyxoviruses (6–8 segments). This correspondence, together with the well-known mapping of the conserved polymerase motifs to the central regions of the L-protein and influenza PB1 subunit, suggests that L-proteins might be architecturally, and functionally equivalent to a concatemer of the three orthomyxovirus polymerase subunits in the order PA-PB1-PB2. Furthermore, our structure of a known influenza endonuclease inhibitor bound to LACV endonuclease suggests that compounds targeting a potentially broad spectrum of segmented RNA viruses, several of which are serious or emerging human, animal and plant pathogens, could be developed using structure-based optimisation.

## Author Summary

Bunyaviruses are a large family of RNA viruses that include serious human, animal and plant pathogens. The viral RNA-dependent RNA polymerase (L-protein) is responsible for replication and transcription of the viral RNA, but apart from its central polymerase domain, it is poorly characterized. Like influenza virus polymerase, bunyavirus L-proteins employ a cap-snatching mechanism to transcribe viral mRNAs, by which host mRNAs are endonucleolytically cleaved as a source of short capped primers. Influenza polymerase endonuclease has recently been located at the PA subunit N-terminus. Here we show biochemically and by crystal structure determination that a similar two-manganese dependent nuclease exists at the N-terminus of La Crosse orthobunyavirus L-protein, whose function is required for cap-dependent transcription. By sequence analysis we show that similar endonuclease signature motifs exist in almost all known segmented RNA, cap-snatching viruses including arenaviruses, bunyaviruses, tenuiviruses and orthomyxoviruses. This suggests that the polymerases of these viruses might share a conserved global architecture with the L-protein being equivalent to a concatenation of the orthomxyovirus PA-PB1-PB2 subunits. We also propose that broad spectrum drugs targeting the endonuclease domain of such viruses could be developed, as exemplified by our structure of the LACV endonuclease complexed with a known influenza endonuclease inhibitor.

## Introduction

*Bunyaviridae* is the largest single family of mostly animal viruses comprising more than 300 species, divided into five genera: Orthobunyavirus, Phlebovirus, Nairovirus, Hantavirus and Tospovirus, the latter infecting plants. The viruses are mainly insect transmitted except Hantaviruses which are rodent borne. They possess a tri-partite negative sense RNA genome, the segments being designated according to size as L, M and S. The L segment encodes a single protein, the RNA-dependent RNA polymerase (polymerase or L protein) which ranges according to genus from 240–460 KDa; the M segment encodes two glycoproteins (Gn, Gc) and in some cases a non-structural protein (NSm) and the S segment encodes the nucleocapsid protein (N) and generally a non-structural protein (NSs). In common with other negative strand RNA viruses, the RNA genome is coated with N protein forming ribonucleoprotein complexes (RNPs) which also contain the polymerase. Bunyavirus particles are generally spherical with the glycoproteins embedded in a membrane envelope which surrounds the RNPs. Replication occurs in the cytoplasm, unlike influenza virus, a negative strand segmented RNA virus of the orthomyxovirus family, which replicates in the nucleus. Bunyaviruses are globally widespread although individual species may be locally restricted by the specificity for particular insect species. Several bunyaviruses are important or emerging human or plant pathogens including La Crosse orthobunyavirus (childhood encephalitis), Hantaan virus (hemorrhagic fever with renal syndrome), Rift Valley fever phlebovirus, tomato spotted wilt tospovirus and Crimean-Congo (hemorrhagic fever) nairovirus.

*Bunyaviridae* polymerases share with those of *Orthomyxoviridae* (e.g. influenza viruses) use of the mechanism of ‘cap-snatching’ for viral mRNA transcription, since, unlike the polymerases from non-segmented negative strand RNA viruses, they do not possess a capping activity. Cap-snatching involves binding of host capped mRNAs to the RNPs, cleavage of these RNAs close to the 5′ cap by a viral endonuclease activity and use of the short capped fragments as primers for viral mRNA transcription. This mechanism was first demonstrated for influenza virus polymerase [1]. An additional 11–15 nucleotides, heterogeneous in sequence, at the 5′ end of the viral mRNA prior to the start of the viral transcribed sequence was observed for snowshoe hare virus [2] and subsequently it was shown that La Crosse virions contain a primer-stimulated RNA polymerase and a methylated cap-dependent endonuclease [3], analogous to the situation found for influenza virus. Subsequently it has been shown that cap-snatching is employed by representative viruses of all five genera of *Bunyaviridae* [4], [5], [6]. *Arenaviridae*, another family of segmented RNA viruses, are also proposed to have a cap-snatching activity [7].

**Figure 1:**
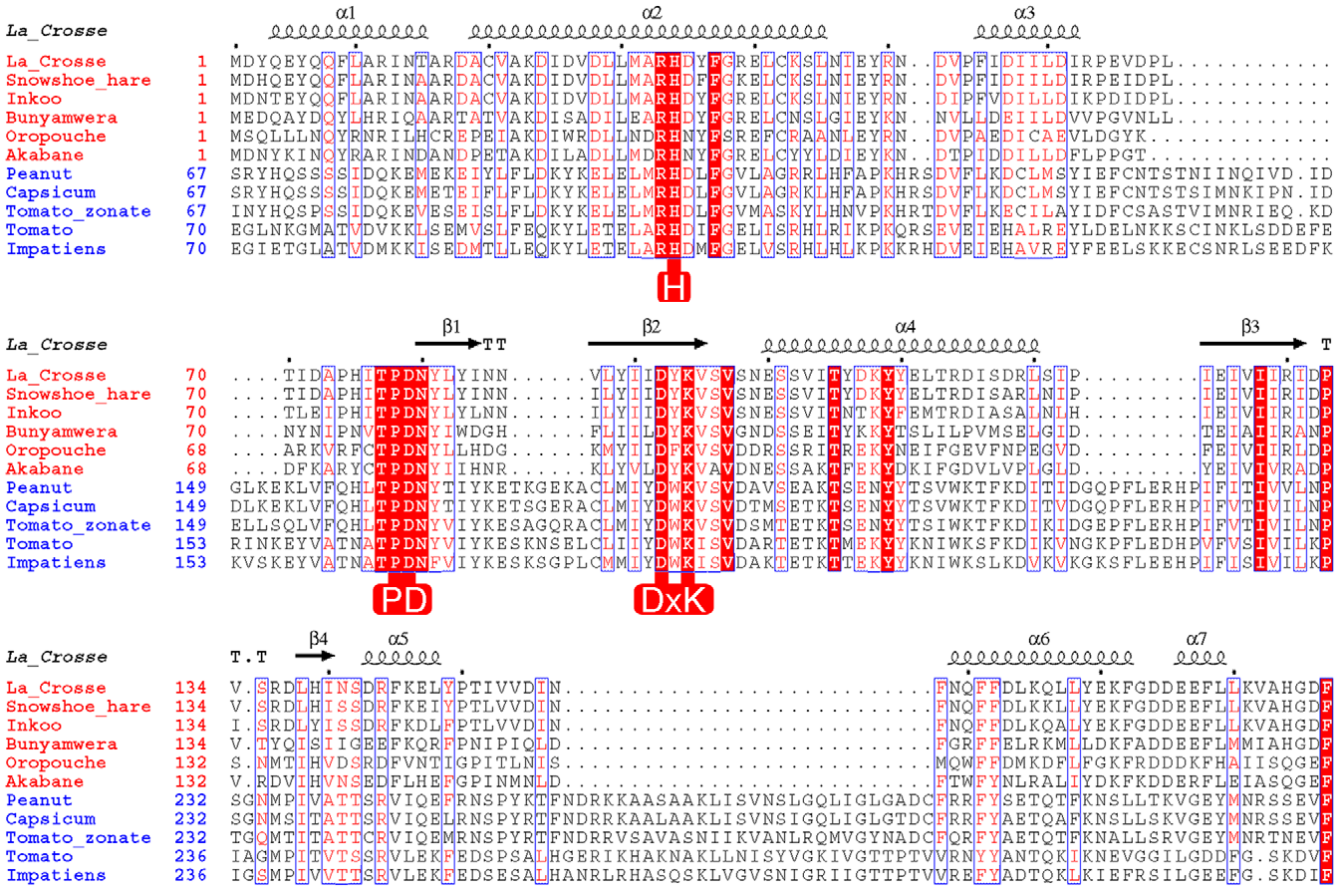
Structure based alignment of the N-terminal domain of Orthobunya (red species names) and Tospovirus (blue species names) L-proteins. The secondary structure derived from the crystal structure of La Crosse virus (LACV) L-protein is shown above the alignment. Key conserved residues important for the nuclease function are highlighted in red.

Although it is well known that the bunya- and arenavirus L-proteins contain in their central region the six polymerase motifs (designated preA, A–E) characteristic of negative-strand RNA viruses [8], [9], [10], the rest of the large protein is completely uncharacterised functionally and structurally, partly due to its lack of sequence homology with other proteins. Recently, crystallographic studies of functional domains of influenza virus polymerase, which is likely to be evolutionary related to the bunyavirus and arenavirus L-protein [8], have precisely defined the location and atomic structure of the two key domains for cap-snatching [11]. The mRNA cap-binding domain is located in the central region of the PB2 subunit [12], whereas the endonuclease activity resides in the N-terminal region of the PA subunit [13], [14]. We therefore asked the question whether this new structural information could aid localisation of domains relevant to cap-snatching in the bunyavirus L-protein? The influenza virus endonuclease domain has a core fold and divalent cation binding residues characteristic of the PD-(D/E)xK nuclease superfamily [15]. Unusually, it has a histidine as one of the metal ligands, which leads to a strong manganese preference for activity [13]. Surprisingly, a highly conserved motif (H....PD...D/E...K) at the extreme N-terminal region of diverse bunyavirus L-proteins with very similar features as now recognised to be important in the influenza PA N-terminal domain, was reported some time ago [8], [16] (Figure 1). This strongly suggested the presence of an endonuclease at the N-terminal of bunyavirus L-proteins. To investigate this further, we used the fact that the influenza endonuclease domain is about 200 residues [13] and made a synthetic gene comprising the first 250 residues of the La Crosse orthobunyavirus (LACV) L protein. Here we present biochemical and structural data that clearly show that the LACV L protein has a functional, manganese-dependent N-terminal endonuclease domain that indeed has very similar characteristics to that of influenza virus endonuclease. We also show that single point mutations that disable the nuclease activity *in vitro*, when introduced into the full-length L-protein, eliminate cap-dependent transcription in a LACV RNP reconstitution assay in cells. By sequence analysis we extend our results to show that all *Bunyaviridae* most likely possess such an endonuclease as well as members of other segmented RNA virus families, such as the bi-segmented *Arenaviridae* and four to six segmented Tenuiviruses. Implications for the evolution of segmented RNA viral polymerases are discussed as well as the prospects for a broad spectrum anti-viral targeting this endonuclease.

## Results

### Crystal structure of the LACV polymerase N-terminal domain

The original 1–250 residue construct of LACV L-protein (LC250) was truncated on the basis of partial proteolysis with papain in order to identify a minimal active and stable fragment that was well expressed and soluble. Papain resistant constructs with C-terminal residue 176, 180, 183, 186 and 190 were produced. The protein encompassing residues 1–180 (LC180) was biochemically characterised and found to be active as a nuclease (see below). The protein encompassing residues 1–183 (LC183) yielded hexagonal crystals which diffract to 2.1 Å resolution, with four molecules per asymmetric unit. The crystal structure was solved by the single anomalous dispersion (SAD) method using seleno-methionine substituted protein. A native data set was refined to an R-factor/R-free of 0.185/0.223 at 2.2 Å. This structure shows clearly one manganese ion bound with octahedral co-ordination in the active site cavity (designated site 1) (Supplementary Figure S1). A second structure, at 2.3 Å resolution (R-factor/R-free = 0.177/0.216), was obtained after soaking the crystals with the diketo acid inhibitor 2,4-dioxo-4-phenylbutanoic acid (DPBA). This is a member of the family of 4-substituted 2,4-dioxobutanoic acids which are known inhibitors of influenza virus endonuclease ([13], [17]). This structure clearly shows in addition to the manganese ion in site 1, a second in an adjacent site 2, with the inhibitor co-ordinating the two ions. The two ions are separated by 3.8 Å and have overlapping octahedral co-ordination (Supplementary Figure S1). In both cases, the identity of the manganese ions was indicated by anomalous scattering (Supplementary Figure S1).

**Figure 2:**
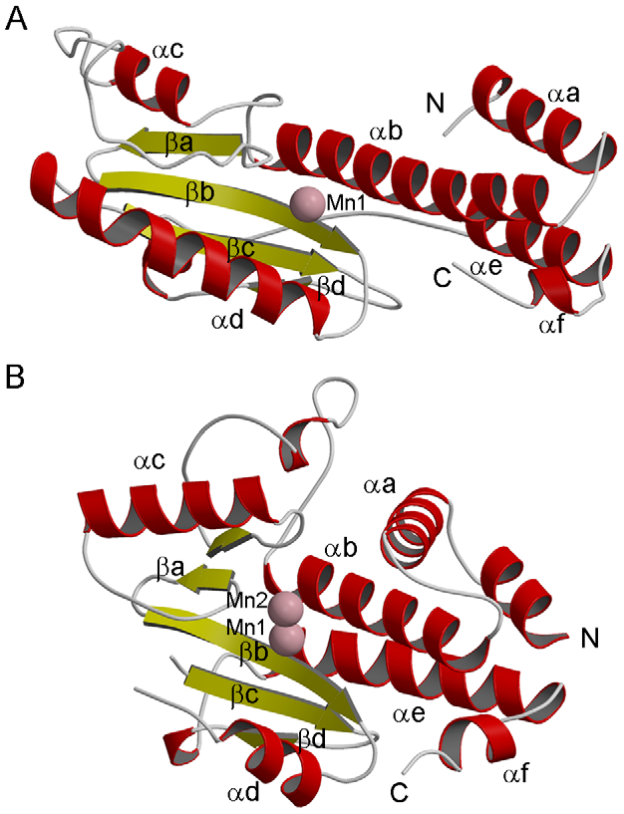
Structure of LC183 compared to influenza A/H3N2 PA-Nter. (a,b) Ribbon diagrams of (a) native LC183 compared with (b) influenza A/H3N2 PA-Nter (PDB entry 2W69) after structural superposition. Helices are in red and beta strands in yellow. Manganese ions are shown as pink spheres. The N and C termini are marked. Corresponding helices and strands in the two structures have the same annotation.

**Figure 3:**
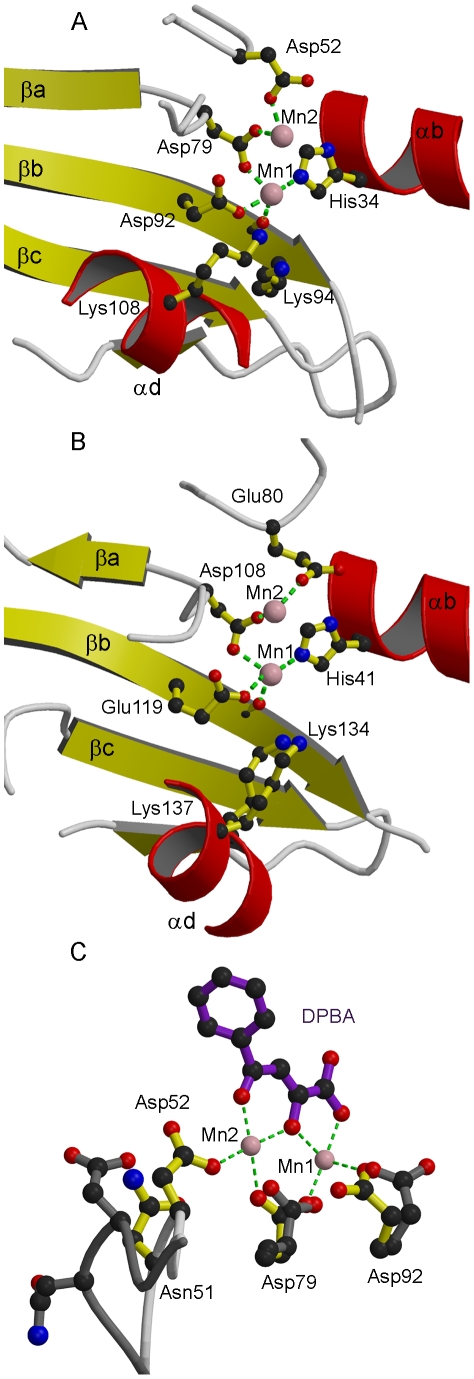
Active site of LC183 compared to influenza A/H3N2 PA-Nter. (a,b) Ribbon diagrams of the active site of (a) DPBA-bound LC183 compared (b) influenza A/H3N2 PA-Nter showing key residues. Annotation and colouring as for Figure 2. Metal co-ordination is shown with a green dotted line (inner sphere water molecules not shown). The catalytic lysine emerges from strand βb in LC183 (Lys94) and from helix αd in PA-Nter (Lys134). Lys108 in LC183 is equivalent to Lys137 in PA-Nter. (c) Induced changes upon binding of DPBA to LC183. DPBA (purple) binds to the two manganese ions (pink spheres) in the active site (yellow side-chains). Binding of the second manganese (Mn2) induces a loop closure allowing Asp52 to become an ion ligand. The superposed native structure without Mn2 bound is drawn in dark grey. Note the change in rotamer of Asp92. See Supplementary Figure S1 for additional details.

The crystal structure of LC183 and its comparison with the N-terminal endonuclease domain of influenza virus polymerase PA subunit (PA-Nter, PDB entry 2W69 [13]) is shown in Figure 2. The secondary structure of LC183, together with a structural alignment of the N-terminal regions of selected orthobunya and tospoviruses L-proteins, is displayed in Figure 1. Comparison of Figures 2a and 2b shows that LC183 has a very similar alpha-beta topology to PA-Nter, although the helices are of significantly different lengths. Notably, the different position of PA-Nter helix αa and the increased length of helix αb gives LC183 a more slender, elongated shape with a more exposed active site that actually lies in a groove between two lobes (Supplementary Figure S2). Focussing in on the active site region, based around a four-stranded anti-parallel beta sheet, the similarity in structure is even more striking (Figure 3), despite essentially no sequence homology. As expected from the initial sequence analysis, LC183 has exactly the same core, cation-binding fold as found in PA-Nter and more generally in the PD-(D/E)xK nuclease superfamily [15]. This core region comprises 55 residues which can be superposed with a root-mean-square deviation of carbon alpha positions of 1.36 Å. Indeed there is a one-to one mapping between the ligands of the two metal binding sites: site 1 has ligands His34, Asp79, Asp92 and the carbonyl-oxygen of Tyr93 in LC183 corresponding to His41, Asp108, Glu119 and Ile120 in PA-Nter; site 2 has ligands Asp52 and Asp79 in LC183 corresponding to Glu80 and Asp108 in PA-Nter. Interestingly the putative catalytic lysine, characteristic of the PD-(D/E)xK nuclease superfamily, is likely to be Lys94 in LC183 (for confirmation, see below). As in *Eco*RV restriction enzyme (see [13] for a comparison of PA-Nter with *Eco*RV), this residue emerges from the central β-strand (βb) of the core fold rather than from helix αd as in the case of PA-Nter (Figure 3). Finally there is a clear correspondence between Lys108 and Lys137 in respectively LC183 and PA-Nter, both emerging from helix αd; in both cases this basic residue is in a position to potentially interact with a nucleic acid substrate. These similarities strongly suggest that LC183 will have a similar two-metal dependent nuclease activity to that of PA-Nter [13].

The inhibitor DPBA binds tightly to the two metal ions in the active site with three of its oxygen atoms replacing three water molecules in the two metal ion co-ordination (Figure 3c, Supplementary Figure S1). The phenyl group of the inhibitor is less well-defined in the electron density indicative of some residual rotational flexibility. This is indicative of the fact that no direct interactions are made between the DPBA and residues of the protein.

Despite the overall high degree of structural similarity of LC183 and PA-Nter, there are some significant differences. In the case of LC183, Asp52, one of the acidic ligands of cation site 2, is on a flexible loop. Indeed in the native structure, this loop is in an open conformation with Asp52 turned away from the active site and consequently only the manganese ion bound in site 1 is present (Figure 3c). In the inhibitor bound structure, the loop is in a closed conformation and Asp52 contributes to binding the second manganese. This suggests that there is preferential and tighter metal binding to site 1, consistent with it having four protein and two water ligands, and weaker metal binding to site 2, which has only two protein oxygen ligands. Furthermore metal binding to site 2 requires closure of the Asp52 loop and may be co-operatively dependent on binding of a nucleic acid substrate or metal binding inhibitor such as DPBA. In contrast, in PA-Nter there is no evidence of flexibility of the corresponding residue Glu80 and PA-Nter can in fact bind a single magnesium atom in site 2 only in the absence of manganese ions [14], [18]. Also, as mentioned above, in PA-Nter, helix α2 and the following loop are positioned to restrict substrate access to the active site cavity, whereas in LC183 the active site opens into a channel which could allow larger, more structured substrates to be cleaved (Supplementary Figure S2). These differences might account for some of the small discrepancies observed in nuclease activity between the two enzymes (see below).

### Divalent cation dependent nuclease activity and thermal stability of the LACV polymerase N-terminal domain

**Figure 4:**
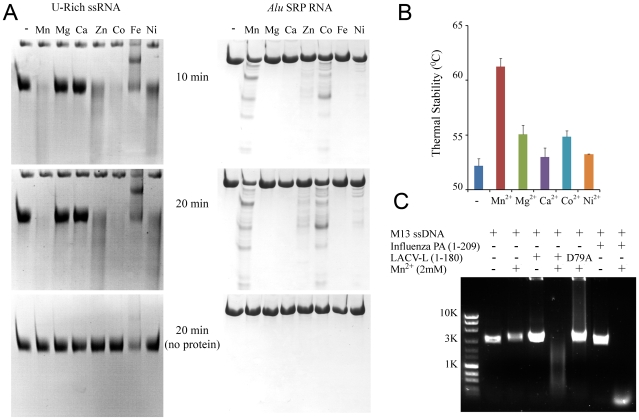
Divalent cation-dependent nuclease activity and thermal stability of LC180. (a) RNA Nuclease activity of LC180. 12 µM of LC180 were incubated at 37°C with 15 µM of U-Rich ssRNA or 12 µM of *Alu* SRP RNA for 10 and 20 minutes with or without 2 mM of the indicated divalent cations. A negative control without protein is shown at 20 minutes of incubation. (b) Thermal stability of LC180. The thermal stability of LC180, with or without 2 mM of the indicated divalent cation, was measured in a Thermofluor experiment [19]. The apparent melting temperatures presented are the average and standard deviations from three different experiments (for raw data, see Supplementary Figure S5). (c) DNA nuclease activity. Manganese dependent nuclease activity on ssDNA for LC180 is shown in comparison with that of influenza A/H3N2 endonuclease (residues 1–209, Dias et al. 2009). 30 µM of protein and 0.75 µg of phage M13 DNA were incubated for 3 hours at 37°C in the presence or absence of 2 mM Mn. The inactive LC 180 mutant (D79A) was included as a negative control.

**Figure 5:**
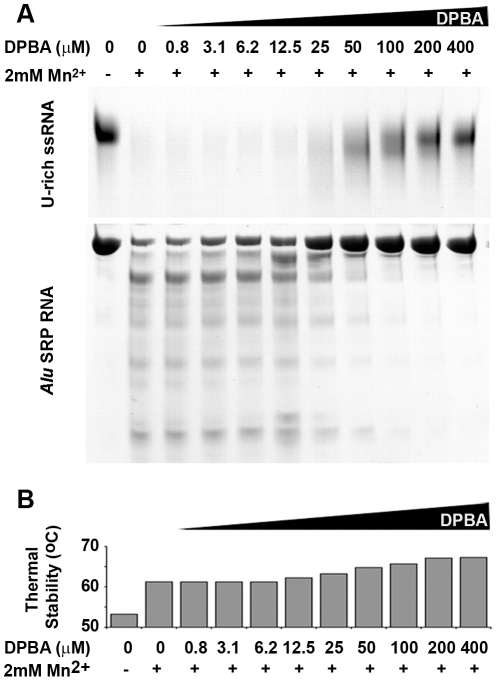
Nuclease inhibition and temperature stabilization of LC180 by 2,4-dioxo-4-phenylbutanoic acid (DPBA). (a) LC180 was incubated at a concentration of 12 µM with 15 µM of U-rich RNA or 12 µM Alu SRP RNA for 20 minutes at 37°C with increasing concentrations of DPBA. The inhibitory effect of DPBA is observed above 12.5 µM and the IC50 is estimated between 25 and 50 µM. (b) The thermal stability of LC180 was measured in a Thermofluor experiment in the same reaction conditions as (a) but without RNA. The inhibitory and thermal stabilising effects of DPBA on LC180 are directly correlated.

Biochemical characterisation of the nuclease activity of LC180 was investigated using RNA and DNA digestion assays in the presence of a variety of divalent metal ions. Because of the structural similarity of the active sites of LC180 and PA-Nter, the experiments were guided by our previous work on influenza PA-Nter and used two of the same RNAs, a single-stranded, unstructured 51 nucleotide (nt) U-rich RNA and a highly structured 110 nt RNA, SRP *Alu* RNA as well as ssDNA [13]. Using 2 mM metal ions, Figure 4a shows that LC180 fully digests the U-rich RNA and partially digests the *Alu* RNA only in the presence of manganese, cobalt, zinc and nickel ions, with the preference Mn>Co≫Zn>Ni and not in the presence of magnesium, calcium or iron. Manganese dependent ssDNA endonuclease activity was observed for LC180 using a circular ssDNA, as for PA-Nter (Figure 4c). Since no digestion of RNAs was observed with 2 mM magnesium ions, increasing amounts of magnesium were tested. Weak nuclease digestion of the U-rich RNA was only observed at very high magnesium concentrations above 12.5 mM (Supplementary Figure S3). We next tested inhibition of the manganese dependent nuclease activity by the diketo acid DPBA. As in the case of PA-Nter, DPBA inhibits digestion of both test RNAs with an estimated IC50 of between 25–50 µM (Figure 5).

In parallel with these nuclease activity tests we measured the metal ion and inhibitor dependent thermal stability of LC180 by a Thermofluor assay [19], again in analogy to previous experiments described for PA-Nter [13]. The metal-free domain has an apparent melting temperature of 52.2 (±0.7)°C. Thermal stability is enhanced by 9°C upon addition of 2 mM manganese, presumably due to metal binding, with smaller increases with magnesium, calcium and cobalt (Figure 4b). A supershift of about 5°C in thermal stability is observed when DPBA is added to LC180 that is pre-bound with manganese and this supershift correlates with the nuclease inhibition affect of DPBA (Figure 5b), strongly suggesting that DPBA binds to the metal ions in the active site, as indeed observed in the crystal structure (Figure 3c).

The relative activity of influenza virus endonuclease (PA-Nter) and LC180 was compared under the same experimental conditions for both test RNAs with 2 mM of manganese, magnesium and calcium. Both enzymes are inactive with calcium, LC180 is more active with manganese and PA-Nter is active with 2 mM magnesium, whereas LC180 is not (Supplementary Figure S4). These experiments highlight three differences between LACV and influenza endonucleases. Firstly, influenza endonuclease is active in the presence of magnesium whereas LACV is not, secondly LACV is more active against the largely double stranded SRP *Alu* RNA and thirdly, LC180 is intrinsically more thermally stable with an apparent melting temperature of 52°C compared to 44°C for PA-Nter [13]. The more efficient activity against structured RNA could be due to the greater accessibility of the active site for LC183 as mentioned above (Supplementary Figure S2). It remains to be investigated whether there are any sequence preferences in the cleavage site favoured by the LACV endonuclease.

### Mutational analysis of the *in vitro* nuclease activity and thermal stability of the LACV polymerase N-terminal domain

**Figure 6:**
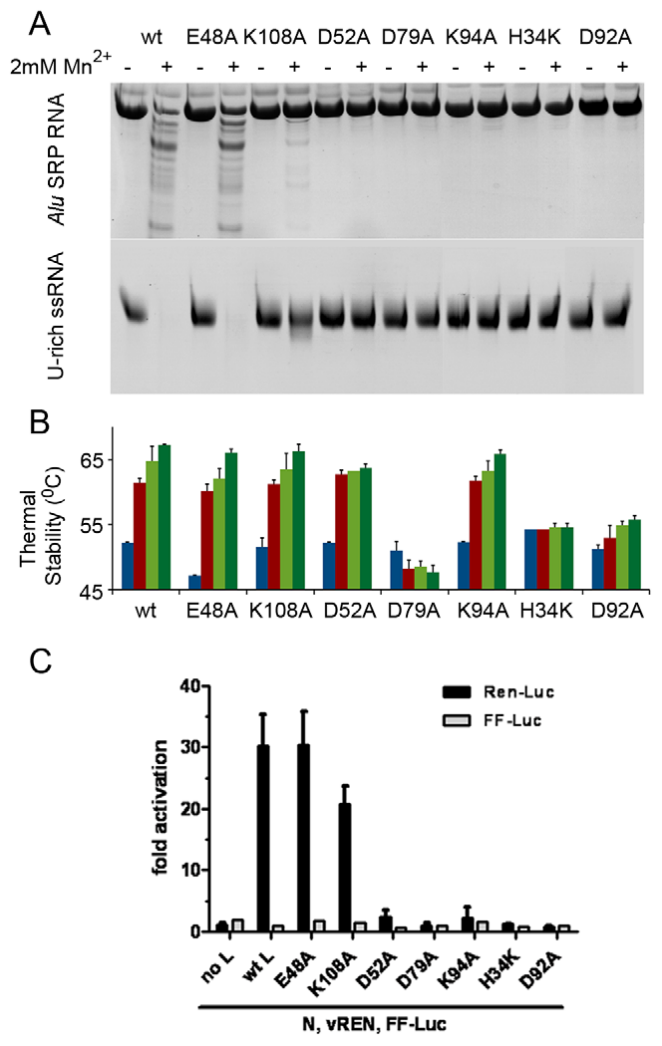
Mutational analysis of the *in vitro* nuclease activity and thermal stability of LC180. (a) Nuclease activity of LC183 mutants. Nuclease activity for each mutant was performed as described using *Alu* SRP (top) or U-rich RNA (bottom) in reactions of 20 minutes, in the presence or absence of 2 mM Mn. (b) Thermal stability of LC183 mutants. Wild-type and mutant LC180 proteins were analyzed in Thermofluor experiments in the absence of divalent cations (blue bars), in the presence of 2 mM Mn (red bars) and in the presence of the 2 mM Mn plus 20 µM or 200 µM DPBA (clear and dark green bars respectively). The data presented are the average and standard deviations of three different experiments. (c) Cap-dependent transcription activity of wild-type and mutant LACV L-proteins. Huh7 cells were transfected with IRES-containing expression plasmids for the LACV nucleocapsid protein (N) and wild-type and mutant constructs for the LACV L protein, as well as with the pol I-driven LACV minireplicon construct containing the REN-Luc reporter gene in negative sense (vREN) [21]. As a negative control the L-protein expression plasmid was omitted. An IRES-containing FF-Luc plasmid was added to the plasmid mix to control expression efficiency. Cell lysates were assayed 24 h post transfection for REN-Luc and FF-Luc activities. Luciferase counts were normalised to the negative control. Mean values and standard deviations from three independent experiments are shown. For controls see Supplementary Figure S8.

We made a series of alanine point mutants of key conserved residues in the active site of LC180 in order to assess their importance for activity. These included the ligands of metal 1 (His34, Asp92 and Asp79) and of metal 2 (Asp79 and Asp52), the putative catalytic lysine 94 and a second lysine (Lys108) close to the active site that is also highly conserved in bunya viruses and in PA-Nter. As a negative control, we also mutated Glu48, again conserved in all orthobunya viruses, which was not predicted from the structure to be directly involved in the nuclease activity. All mutant proteins were well expressed as for wild-type and purified as folded proteins as judged by behaviour on gel-filtration and in thermal stability assays (Figure 6). Only the H34A mutated protein was found to be somewhat less temperature stable, probably due to a loss of charge complementation in the highly acidic active site. A H34K mutant was made and assayed instead. The results of nuclease assays with these mutant proteins with the two RNAs and of thermal stability assays are shown in Figure 6a and 6b respectively. Mutations of any single of the four metal binding ligands (H34K, D52A, D79A and D92A) leads to elimination of nuclease activity, as does mutation of the catalytic lysine (K94A). The mutation E48A has no effect on activity and the mutation K108A leads to a reduction in activity, possibly because loss of the positively charged side-chain reduces substrate RNA binding. As described above, the wild-type protein shows significantly higher temperature stability upon binding of 2 mM manganese which is reinforced by subsequent binding of the inhibitor DPBA (Figure 4, Figure 5). Essentially the same pattern is shown by the mutants E48A (negative control, which however has somewhat reduced protein stability), K108A and K94A. This is consistent with the structural information which shows that none of these residues are directly involved in metal ligation or inhibitor binding. The D52A binding retains the manganese effect but has no inhibitor effect. Since D52 only binds metal 2, and as our structures show, a single manganese can bind strongly to metal site 1 (with the D52 loop in an open conformation, Figure 3c), we interpret this to imply that a single manganese ion bound in site 1 is sufficient to give the enhanced stability effect, whereas one ion is not sufficient to bind the inhibitor DPBA. The mutant D79A has no enhanced stability (in fact slightly reduced stability) in either the presence of manganese ions, with or without inhibitor, consistent with the fact that it binds simultaneously both metal ions. The H34K mutant has slightly increased stability compared to wild-type, probably because the lysine side-chain charge compensates the acidic active site better than the histidine, but there is no effect of manganese or inhibitor. This mutation thus almost certainly prevents any metal binding. Finally the mutation D92A shows a very modest manganese and inhibitor effect. It is thus possible that this mutant has still some low affinity for both metals. In summary these mutational studies show that the nuclease activity of LC180 depends critically on an intact binding site for two metal ions, preferably manganese, as well as the presence of the catalytic Lys94. Furthermore, binding of only one manganese ion is sufficient to lead to enhanced thermal stability, whereas both metal ions are required for DPBA binding. These results are fully consistent with the crystal structures described above.

To quantify the thermodynamics of binding of manganese to LC180 we used isothermal titration calorimetry in which manganese ions were titrated into wild-type or D52A mutant LC180 (see methods and Supplementary Figure S6). For wild-type protein the ITC data were fitted with a model comprising two independent sites yielding Kd's of 7.20 (±1.73) and 159.0 (±42.9) µM, although in the experiment saturation of the weaker binding site was not achieved. For the D52A mutant the ITC data were satisfactorily fitted with a model comprising a single site giving a Kd of 21.0 (±2.3) µM, with saturation of the single site being achieved. More complete results for the thermodynamic parameters of manganese binding are given in Supplementary Table S1. Once again these results are fully consistent with our structural and thermal stability experiments with the interpretation that the strongly bound ion for the wild-type and the single site for the D52A mutant (which have comparable affinities) corresponds to metal site 1 and the more weakly bound site for the wild-type corresponds to metal site 2. When magnesium was substituted for manganese no binding was detected by ITC. An analogous mutational and quantitative metal binding analysis has recently been performed for influenza virus endonuclease [20], with slight differences in behaviour being observed, as mentioned above.

### Mutational analysis of the transcriptional activity of the full-length LACV polymerase

To test the effect of the nuclease inactivating mutants in the context of the full-length LACV L-protein we used a previously described *in vivo* RNP reconstitution system in which a *Renilla* Luciferase (REN-Luc) reporter gene is used as a readout of cap-dependent transcription by the viral polymerase [21] (For a schematic outline of this assay see Supplementary Figure S7). From the *in vitro* work we know that the mutations do not disrupt the folding of the endonuclease domain and therefore presumably not of the full-length L-protein. Moreover, expression levels of full-length wild-type and mutant L constructs are comparable as detected by immunofluoresence (Supplementary Figure S8a). The transcription assay results with the various mutants (Figure 6c) parallel very closely the *in vitro* nuclease activity of the isolated LC180 domain mutants. Only the wild-type, negative control (E48A) and K108A (slightly reduced activity) L proteins give rise to significant REN-Luc production. To detect whether these active mutants are indeed producing capped mRNAs, we co-expressed them with the polio virus 2A^pro^ protein. This protease specifically abrogates cap-dependent mRNA translation by cleaving eukaryotic initiation factor (eIF)4G [22]. The T7-driven expression constructs for the LACV L and N proteins, as well as the firefly luciferase (FF-Luc) transfection control escape this inhibition, since their translation is mediated by a viral internal ribosome entry site (IRES). As shown in Supplementary Figure S8b the Ren reporter activity of all active LACV L variants is drastically reduced upon co-expression of 2A^pro^, whereas the 2A^pro^ mutant G60A, which has lost eIF4G cleaving activity [22], had no such effect. Moreover, IRES-driven FF-Luc expression was not affected by 2A^pro^, as expected (Supplementary Figure S8c). Thus, the specific sensitivity of L-protein driven Ren activity to the polio virus 2A^pro^ indicates that wt L and both the E48A and the K108A mutant transcribe capped mRNAs.

Taken together, these results show that cap-dependent transcription is absolutely dependent on a functional two manganese-dependent nuclease activity at the N-terminus of the LACV L-protein, strongly suggesting that this domain is the cap-snatching endonuclease of the viral polymerase.

## Discussion

Cap-snatching as a method of priming transcription is uniquely restricted to segmented negative strand viruses, notably orthomyxoviruses (influenza), bunyaviruses and arenaviruses. The recent structural characterisation of two functional domains relevant for cap-snatching by influenza polymerase, the cap-binding domain and the endonuclease, in respectively the PB2 and PA polymerase subunits, raise the question as to whether similar domains exist in the L-protein (polymerase) of bunya- and arenaviruses. The work presented here shows unequivocally that the extreme N-terminal 200 residues of LACV has a cap-snatching endonuclease activity with very close structural and biochemical features to that of the N-terminal domain of the influenza virus polymerase PA subunit. We do not yet know the context of the bunyavirus N-terminal endonuclease within the 3-dimensional structure of the complete polymerase. However it is likely that there is a cap-binding domain and probably other RNA binding domains within the polymerase (this is certainly true for influenza polymerase) that enhance affinity and provide specificity for capped cellular mRNAs. Also it is possible that, as with influenza virus, there are allosteric effects that activate or make accessible the endonuclease active site only upon cap-binding.

**Figure 7:**
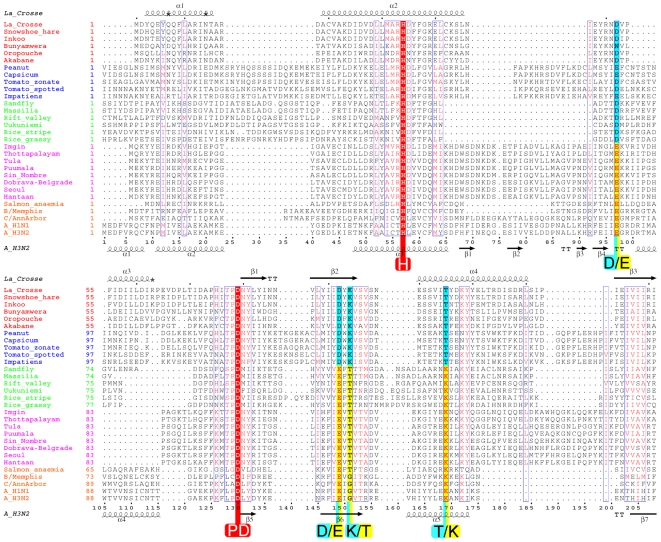
Structure based multiple alignment of the endonuclease of four genera of bunyavirus L-proteins together with the endonuclease of the PA subunit of selected orthomyxoviruses. Sequences from selected species of orthobunyaviruses (red names), tospovirus (blue), phlebovirus (green), hantavirus (purple) and orthomyxoviruses (orange) were aligned using CLUSTALW [49] and adjusted manually based on the known structural alignment of LACV (top secondary structure) and influenza A/H3N2 (bottom secondary structure). Rice-stripe (Acc. No. ABC68333, [26]) and rice-grassy stunt viruses (Acc. No. NP_058528, [51]) are actually classified as tenuiviruses [23] but have L-proteins with similar features to phleboviruses. The key cation binding residues and catalytic lysine are indicated with large letters. Universally conserved residues are in red. The key residues occur in two patterns: orthobunya- and tospoviruses have the motif H....D...PD....DxK.....T (cyan colours) and hanta- and orthomyxoviruses have the motif H....E...PD....ExT.....K (yellow colours). Phlebo- and tenuiviruses have the second motif but with an aspartate in the second position. The catalytic lysine switches between two positions, exchanging with a conserved threonine (except in orthomyxoviruses). The orthomyxoviruses with a clearly defined endonuclease motif include influenzas A, B and C and salmon anemia virus (Acc No. ABF68032). Other orthomyxoviruses have minor (Quaranfil virus, Acc. No. ACY56279, [32]) or significant (Thogoto virus, Acc. No. AAB62893) deviations in the endonuclease motif at the N-terminus of the PA subunit (data not shown). For a discussion of nairoviruses, other unclassified bunyaviruses and arenaviruses, see text and Figure 8.

**Figure 8:**
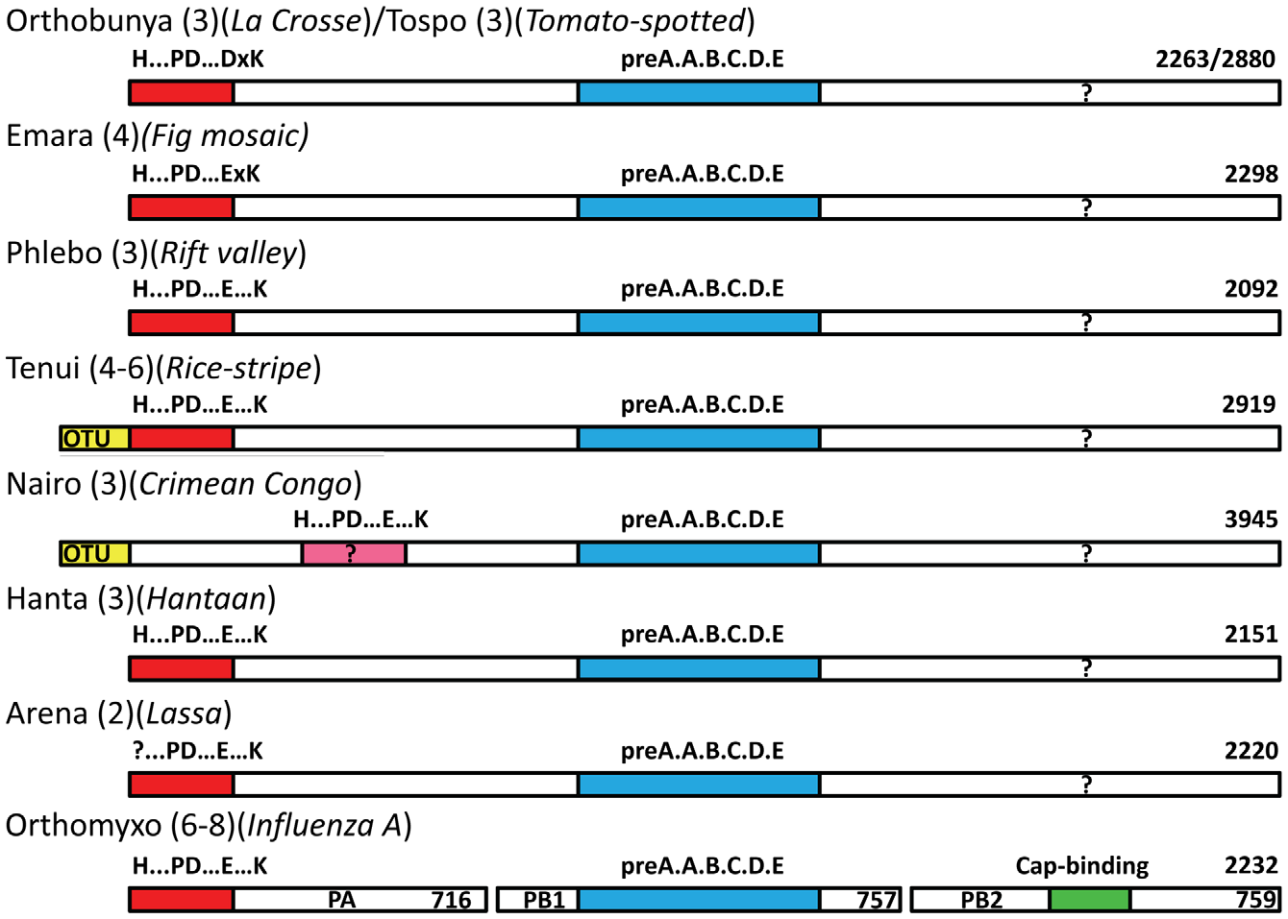
Schematic diagram of the polymerase architecture of negative strand segmented viruses. The polymerase proteins of different genera of negative strand segmented RNA viruses known or suspected to use cap-snatching are represented as bars (not drawn to scale). In brackets is indicated the number of genome segments and an example species, the length of whose polymerase in amino acids is indicated at the right hand end. The red bar at the left indicates the endonuclease domain with the particular sequence signature of that genus written above. For Crimean Congo virus this is in pink due to the higher uncertainty of the assignment. The blue bar represents the conserved polymerase domain in the central region. In the case of orthomyxoviruses, the three subunits PA, PB1 and PB2 are represented co-linearly. The green bar in PB2 represents the cap-binding domain, which is possibly located in the C-terminal region of the L-proteins (question mark).

We next examined whether the endonuclease signature could be identified in the L-protein of other segmented RNA viruses. Sequence analysis gives strong evidence that a homologous endonuclease domain exists at the N-terminus of the L-protein of four *Bunyaviridae* genera, orthobunya-, tospo, phlebo and hantaviruses, as well as tenuiviruses (which have four to six genome segments, [23], http://www.ncbi.nlm.nih.gov/ICTVdb/ICTVdB/00.069.0.01. Tenuivirus) and orthomyxovirus (Figure 7). In each case, the key metal binding and catalytic lysine residues can be identified. The sequence analysis shows that there are two sub-groups of these enzymes, with slightly different endonuclease signatures. Orthobunya- and Tospoviruses have the motif H....D...PD....DxK.....T, whereas Phlebo- and Hantaviruses have the motif H....E...PD....ExT.....K (although in Phleboviruses the first E is replaced by a D). The Hantavirus motif is identical to that found in orthomyxoviruses (Figure 7). The first version has a preference for aspartates and the catalytic lysine emerges from beta-strand βb, whereas the second version has a preference for glutamates and the catalytic lysine emerges from alpha helix αd (see Figure 3ab). Interestingly, the catalytic lysine interchanges with an absolutely conserved threonine at the two alternative positions (Figure 7). Nairoviruses are not included in this alignment as the location of the endonuclease is less certain. This genus of *Bunyaviridae*, which includes Crimean-Congo hemorrhagic fever virus, has an unusually long L-protein (about 4000 residues, compared to 2100–2900 for most other bunyaviruses). The N-terminal half of nairovirus L-proteins (i.e. prior to the polymerase motifs which start around residue 2050) contains a putative ovarian tumour (OTU)-like cysteine protease at the beginning [24], [25] as well as other predicted motifs and domains [10]. A putative endonuclease motif of the Phlebo/Hanta/Orthomyxo type exists in the residue range 630–710 (H(632)...PD(672)....E(686)F....K(699), numbering for Crimean-Congo virus) [10], but this needs to be confirmed by structural and functional data. It is interesting to note that the rice stripe tenuivirus also contains a predicted N-terminal OTU-like protease before the endonuclease motif [26]. It has been suggested that the protease might release the viral polymerase and one or more additional proteins by autoproteolytic cleavage and/or have de-ubiquitination activity [26]. Indeed de-ubiquitination activity of Crimean-Congo virus OTU domain has been shown to inhibit Ub- and ISG15-dependent antiviral pathways [27]. Arenavirus L-proteins have a highly conserved N-terminal region of about 200 residues that contains the absolutely conserved sequence of residues PD(89)...E(102)xF....K(122)L (alignment not shown, numbering for Lassa virus). This closely resembles the Phlebo/Hanta/Orthomyxo endonuclease motif, although the histidine is clearly lacking. Very recently, systematic alanine mutation of conserved charged residues in Lassa virus L-protein outside the polymerase motifs have been performed and the effect on transcription and replication have been tested in a RNP reconstitution system [28]. Seven charged residues in the N-terminal region, including Asp89, Glu102 and Lys122 and Asp129, were selectively important for mRNA synthesis but did not affect genome replication. The authors concluded from these results, combined with sequence similarities to type II endonucleases and influenza virus endonuclease, that this region of the L-protein was likely to be the cap-snatching endonuclease of arenaviruses, in full agreement with our analysis. Finally, the endonuclease signature is also clearly present in the L-proteins of two related but unclassified bunyaviruses (proposed to be called emaraviruses) which have four rather than the usual three genome segments, European mountain ash ringspot disease (Acc. No. YP003104764, [29]) and fig mosaic virus (Acc. No. CAQ03479, [30]). Both have the motif RH(105)D...PD(144)...E(158)xK(160) (numbering for mountain ash ringspot disease virus) and are thus most closely related to the Orthobunya and Tospoviruses, All these observations are summarised in Figure 8 which shows a schematic diagram of the architecture of polymerases from negative strand segmented RNA viruses.

It is well known that the 6 motifs characteristic of negative strand RNA-dependent RNA polymerases (pre-motif A and motifs A–E) are present in the central region of bunya and arenavirus L-proteins and in the PB1 subunits of orthomyxoviruses [8], [9], [10]. The current work shows that the extreme N-terminal region of bunya-, tenui- and arenavirus L-proteins functionally corresponds to the N-terminal region of the PA subunit of orthomyxoviruses. Given that the three influenza A polymerase subunits total 2252 residues, very similar to the size of many bunyavirus complete L-proteins and all these viral enzymes have common mechanisms of transcription (cap-snatching) and replication, a natural hypothesis that follows is that the L-proteins might be architecturally, structurally and functionally equivalent to a concatemer of the three influenza polymerase subunits in the order PA-PB1-PB2 (Figure 8). Some indirect support for the functional concatenation of the influenza polymerase subunits comes from the fact that the inter-subunit interactions are dominated by contacts between the C and N-terminal extremities of respectively PA and PB1 and PB1 and PB2 as visualised by recent crystal structures (reviewed in [11]). The most significant implication of this hypothesis is that the C-terminal third of the L-protein might be structurally and functionally equivalent to PB2, which contains the cap-binding domain required for cap-snatching. Unfortunately, this region of the L-protein is the least well conserved and there are no obvious cross-genera conserved motifs that could point to a putative cap-binding site similar to that described for influenza A PB2 subunit [12]. This is perhaps not surprising as the PB2-like subunits of, for instance, salmon anaemia and Quaranfil viruses, two non-influenza orthomyxoviruses, are highly diverged from influenza [31], [32], even though both these viruses appear to possess an endonuclease at the N-terminus of the PA subunit (Figure 7). Furthermore the fact that the distance of endonucleolytic cleavage from the 5′ cap is rather variable amongst cap-snatching viruses [6] suggests that the location of the cap-binding domain might vary. In fact, there is no clear proof that any L-protein directly binds capped RNAs and even some evidence that in hantaviruses the viral N-protein may play this role [33]. Clearly more experimental work is required to elucidate the complete mechanism of cap-snatching in bunya-, tenui- and arenaviruses and to validate or otherwise the hypothesis that L-proteins are architecturally equivalent to the concatenation of PA-PB1-PB2.

Finally it is important to note that for nearly two decades, influenza virus endonuclease has been targeted for anti-viral drug discovery and a number of specific endonuclease inhibitors have been described [17], [34], [35], [36], [37]. Most of these compounds implicitly target the two metal binding site of the endonuclease, which is also the target for many HIV integrase inhibitors [38] including the currently approved raltegravir [39], [40]. The recent structure determination of the endonuclease of influenza virus polymerase [13], [14] gives new impetus to structure-based optimisation of these inhibitors. The results described here show that bunyaviruses and arenaviruses, amongst which are several dangerous and emerging pathogens, contain a very similar endonuclease to influenza virus, which is also therefore a good target for anti-viral drug design. Indeed, the close similarities between influenza and bunyavirus endonucleases suggests that compounds targeting a broad spectrum of segmented negative strand RNA viruses could be envisaged. Our structure of DPBA bound to LACV endonuclease shows that this is indeed the case, although this compound is of low potency [17]. In addition this structure provides the first concrete proof that these compounds do indeed chelate the two divalent cations in the endonuclease active site.

## Methods

### Plasmids

The coding sequence of the N-terminal 250 residues of LACV-L (LC250) (UniProt accession code A5HC98) was optimised for expression in *E. coli* and synthesized (Geneart). A histidine tag followed by a linker and a TEV cleavage site (MGHHHHHHDYDIPTTENLYFQG) was added to the amino terminus of all protein constructs. All protein variants were amplified by PCR and cloned into a pET9a (Novagen) modified vector between *NdeI* 5′ and *NotI* 3′ sites for expression in *E. coli*. Mutagenesis of the proteins expressed in *E. coli* was performed on LC180. Mutant constructs were obtained by site directed mutagenesis using overlapping oligonucleotides and *Pfu* or KOD (Novagen) DNA Polymerases. The constructs pTM-LACV-L, pTM-LACV-N, pLACV-vRen, pCAGGs-T7 and pTM-FF-Luc used in the RNP reconstitution have been described previously [21]. The pTM1-based expression constructs for poliovirus 2APro wt and G60R mutant were kindly provided by Luis Carrasco, Universidad Autónoma de Madrid, Spain [22]. Mutagenesis of the cDNA for the RNP reconstitution experiments was performed by generating mutant DNA fragments by PCR and insertion into the *Kpn*I/*Bmt*I restriction sites of the pTM-LACV-L vector. In all cases the correctness of the DNA constructs were confirmed by DNA sequencing.

### Protein expression and purification

Proteins were expressed in *Escherichia coli* strain BL21 (DE3) in LB media with 25 µM kanamycin at 18°C overnight after induction with 0.2 mM of IPTG. Labelled protein was obtained by expressing LC183 protein in *E. coli* with M9 minimal medium and 50 mg/L of seleno-methionine. The cells were disrupted by sonication on ice for 3 minutes in lysis buffer (20 mM Tris-HCl pH 7.6, 150 mM NaCl, 2.5 mM β-mercapto-ethanol) with EDTA-free protease inhibitor cocktail (Roche). The protein from the soluble fraction was loaded onto a 5 ml Nickel column, washed with 10 volumes of lysis buffer with 50 mM imidazol and eluted with 5 volumes of 400 mM imidazol. The eluted protein was cleaved with histidine tagged TEV protease overnight at 4°C in dialysis against lysis buffer. After TEV cleavage all proteins have an additional glycine at the N-terminus. A second nickel column step was performed to remove unwanted material. The resulting untagged proteins were concentrated and purified by gel filtration chromatography using a SD75 column (Pharmacia) with lysis buffer for *in vitro* experiments or 20 mM HEPES pH 7.6, 150 mM NaCl, 2.5 mM β-mercapto-ethanol for crystallization trials. Influenza A/H3N2 endonuclease (PA 1–209) was obtained as described [13]. Purified LC proteins are contaminated with a small percentage of a degradation fragment of size LC163.

### Identification of the amino terminal domain of LACV-L protein

The length of the proteolytically stable amino terminal domain was defined from the LC250 purified protein by limited papain digestion with 1∶500 (w∶w) papain: protein ratio. Products were characterized by N-terminal sequencing and mass spectrometry. The resulting papain resistant fragments had molecular weights between 20.7 and 21.2 KDa corresponding to the first 175–178 residues of the LACV-L protein. Proteins LC176, 180, 183, 186 and 190 were subsequently produced. Finally, the protein construct LC180 was used for all *in vitro* biochemical experiments and LC183 for structural studies.

### Thermal stability experiments and endonuclease activity assays

The influence of metal ion and DPBA binding on protein stability was measured by Thermofluor assays [19] at a protein concentration of 25 µM in lysis buffer and 2 mM concentration of various metal ions. For nuclease activity experiments, 12 µM of LC180 wild type and mutant proteins were incubated with 12 µM of *Alu* RNA (110 nucleotides of the *Alu* domain of *Pyrococcus horikoshii* SRP RNA) or 15 µM of 51 nucleotides U-rich RNA (5′-GGCCAUCCUGU_7_CCCU_11_CU_19_-3′) [13] at 37°C in the same buffer. The reaction was stopped by adding EGTA at a final concentration of 12 mM. Divalent cations were added to 2 mM final concentration. The reaction products were loaded onto 8 M urea, 15% acrylamide, Tris-borate gels and stained with methylene blue.

### Isothermal titration calorimetry (ITC)

ITC experiments were performed using a high-precision VP-ITC titration calorimetric system (Microcal Inc., Northampton, MA). Binding experiments were performed with 60 µM of freshly purified LC180 protein at 25 C in the same buffer used for the nuclease activity assays. Titrations were made by injecting 15 µl of 1.8 mM or 3 mM MnCl_2_ into the LC180 D52A or wt respectively. For data analysis the heat produced by the metal ion dilution into the buffer was subtracted from the heat obtained in the presence of protein. The same procedure was performed with up to 12 mM of MgCl_2_ but gave no interaction signal. The binding isotherms were analyzed by non-linear least squares fitting (Microcal Origin software) using models corresponding to a single site or two independent sites for the D52A and the wt respectively. Thermodynamic values given are the average and standard deviation of at least two experiments.

### Crystallization

Proteins LC176, 180, 183, 186 and 190 were expressed and tested for crystallization using a Cartesian nanovolume robotic system for screening. Only LC183 and LC186 crystallised and LC183 was used for all subsequent work. Crystals were obtained by mixing 1∶1 ratio protein: reservoir solution of 15–20 mg/ml LC183 protein in 20 mM HEPES pH 7.5, 150 mM NaCl, 5 mM MnCl_2_ and 2.5 mM β-mercapto-ethanol, and a reservoir composition of 3.4 M sodium formate, 0.1 M Tris-HCl at pH 8. The seleno-methionine LC183 crystals were obtained with a reservoir composition of 3.6 M Na-formate, 0.1 M HEPES pH 7. The dataset of the inhibitor-endonuclease complex was obtained after an overnight soaking of native crystals into reservoir buffer with 5 mM MnCl_2_, 10 mM MgCl_2_ and 5 mM of DPBA. The crystals were frozen in liquid nitrogen in the reservoir buffer with 30% glycerol for the selenomethionine labelled protein and with 30% glycerol, 5 mM MnCl_2_, 10 mM MgCl_2_ and 5 mM of DPBA for the inhibitor complex.

### Crystallography

Crystals are of space-group *P*6_1_22 with four molecules in the asymmetric unit. Selenomethionine derivative data were collected on a 180×160×140 µm^3^ crystal to 2.1 Å resolution on beamline ID29 at the European Synchrotron Radiation Facility (ESRF) at the selenium edge (X-ray wavelength 0.979 Å) for experimental phasing. Native and DPBA data were collected to 2.2 Å resolution on ID29 with wavelengths of 0.954 Å and 0.976 Å respectively. Data were processed and scaled with the XDS package [41] and subsequent analysis performed with the CCP4i package. Statistics of data collection and refinement are given in Table 1. The structure solution was obtained by the SAD method using autoSHARP [42] which found 16 anomalous sites, four (including a manganese site) for each of the four chains in the asymmetric unit. The resultant map was excellent and could be largely built automatically by ARP/wARP [43]. Refinement was performed with REFMAC [44] without applying non-crystallographic symmetry restraints. Extra density was observed for a single Mn^2+^ ion in the active site of each of the four molecules in the asymmetric unit as confirmed by strong anomalous scattering, even though the X-ray energy was well away from any manganese edge (Supplementary Figure S1). The loop containing Asp52 is either in the open position or partially open and intermediate. The structure of the complex with the inhibitor was solved by molecular replacement using PHASER [45] and the previously obtained model. Extra density was observed for a second Mn^2+^ and for the DPBA (Supplementary Figure S1). The loop containing Asp52 is in the closed position.

**Table 1:** Data collection and refinement statistics.

	SeMet proteinOne manganese	DPBA-boundTwo manganese	Open loopOne manganese
Space Group	*P*6_1_22	*P*6_1_22	*P*6_1_22
Cell dimensions (Å)	a = b = 124.53c = 295.20α = β = 90° γ = 120°	a = b = 124.61c = 294.74α = β = 90° γ = 120°	a = b = 124.20c = 294.32α = β = 90° γ = 120°
Resolution range (last shell) (Å)	50−2.1 (2.18−2.10)	50−2.20 (2.28−2.20)	50−2.2 (2.3−2.2)
Beamline	ESRF ID29	ESRF ID29	ESRF ID29
Wavelength (Å)	0.9790	0.9763	0.9537
Detector	ADSC Quantum Q315r	ADSC Quantum Q315r	ADSC Quantum Q315r
Completeness (last shell) (%)	100 (100)	100 (100)	95.4 (95.7)
R-sym (last shell)	9.4 (68.1)	8.4 (59.2)	8.0 (59.4)
I/σI (last shell)	13.46 (2.52)	23.4 (5.1)	23.5 (4.2)
No. of reflections used (free reflections)	76324 (3174)	66523 (2787)	62367 (3290)
R-factor (last shell)	-	0.188 (0.232)	0.192 (0.262)
R-free (last shell)	-	0.221 (0.285)	0.224 (0.297)
Total atoms in structure	-	6736	6681
Protein atoms	-	6227	6212
Ligand atoms	-	8xMn^++^, 4xDPDA (56)	4 Mn^++^
Water molecules	-	445	465
Average B-value (Å^2^)	-	26.1	25.8
Ramachandran plot favoured regions	-	98.7	98.9
Ramachandran plot allowed regions	-	100	99.9
Bond distance deviations from ideal (Å)	-	0.018	0.018
Angles deviations from ideal (°)	-	1.56	1.55

### LACV RNP reconstitution system

Sub-confluent monolayers of Huh7 cells in 12-well plates were transfected with 0.25 µg each of pLACV-vREN and pCAGGs-T7, 0.4 µg of pTM-LACV L (wild type or mutants) and pTM-LACV N, and 0.1 µg of pTM-FF-Luc using Nanofectin transfection reagent (PAA). In the negative control, the LACV-L expression plasmid was omitted from the transfection mix. An additional 0.2 µg of empty vector pTM1, or expression constructs pTM1-2A^Pro^ or pTM1-2A^Pro^(G60R) were transfected in some experiments, as indicated. After transfection, cells were incubated for 24 h and lysed in 100 µl Dual Luciferase Passive Lysis Buffer (Promega). An aliquot of 20 µl of the lysate was assayed for FF-Luc and Ren-Luc activities as described by the manufacturer (Promega).

### Software

Structure figures were drawn with Molscript [46] or Bobscript [47] and rendered with Raster3d [48]. Sequence alignments were performed with ClustalW [49] and drawn with ESPript (http://espript.ibcp.fr/ESPript/cgi-bin/ESPript.cgi) [50]. Molprobity was used to analyse the quality of the structures (http://molprobity.biochem.duke.edu/).

### Co-ordinates and structure factor deposition

The native structure of LC183 has wwPDB ID 2xi5 for the coordinate entry and r2xi5sf for the structure factors. The DPBA-bound form of LC183 has wwPDB ID 2xi7 for the coordinate entry and r2xi7sf for the structure factors.

## Supporting Information

Figure S1 Metal ion and inhibitor binding in the active site of LC183. **(a)** Native LC183 structure with only one ion manganese bound. Blue: final 2fo-fc electron density at 0.95 σ (blue net). Anomalous difference map contoured at 4.0 σ (yellow net). Manganese ions and water molecules are represented by pink and blue spheres respectively. Mn1 has anomalous peak heights of between 7.3 and 11.5 σ for the four independent molecules in the asymmetric unit. **(b)** DPBA bound structure with two manganese ions. DPDA is in purple stick representation. Colours are as in **(a)** with in addition, the unbiased fo-fc positive difference electron density at 0.95 σ (brown net). Mn1 and Mn2 have anomalous peak heights of respectively between 17.8 and 20.9 σ and between 10.3 and 11.8 σ, for the four independent molecules in the asymmetric unit. **(c)** Diagram showing full cation and DPBA co-ordination (green dotted lines).
(1.55 MB TIF)

Figure S2 Active site accessibility of the LACV and Influenza virus endonuclease structures. Surface representation of the LACV (top, cyan) and Influenza (bottom, violet) endonuclease structures in two orientations after superposition. The conserved active site residues are coloured in yellow and the two metal ions are red spheres. The LC183 active site is in an open channel formed between two lobes of the protein into which double-stranded nucleic acid might fit. In contrast the PA-Nterm active site is less accessible in the bottom of a deep depression cavity with an estimated volume of 536 Å^3^ (programme POCASA). N- and C-termini of the proteins are marked.
(1.93 MB TIF)

Figure S3 Nuclease assays of LC180 in the presence magnesium. Increasing concentrations of magnesium ions were tested in endonuclease activity assays with 6 µM of protein concentration and 7.5 or 6 µM concentration of U-rich or *Alu* SRP RNAs respectively. Significant RNA digestion is only observed above 12.5 mM concentration of magnesium. Even at the highest concentrations the activity with magnesium is far less than that with 2 mM manganese.
(0.83 MB TIF)

Figure S4 Divalent cation dependent nuclease activity of LC180 (LC) in comparison with the influenza endonuclease PA-Nterm (IV). Reactions were carried out with 10 µM concentration of protein and 12.6 µM or 10 µM concentration of U-rich and *Alu* SRP RNAs respectively for 50 min at 37°C in the presence of 2 mM manganese, magnesium or calcium. The maximum activity is achieved with manganese ions for both proteins, although the influenza endonuclease has some activity in the presence of magnesium ions, whereas LC180 does not. Neither have activity in the presence of calcium ions. The LACV protein has higher activity with the highly structured Alu SRP RNA than the influenza endonuclease.
(4.79 MB TIF)

Figure S5 Single Thermofluor experiment of LC180 in the presence of 2 mM of various divalent cations. Thermofluor experiments were performed as described in the methods (Ericsson UB, Hallberg BM, Detitta GT, Dekker N, Nordlund P (2006) Thermofluor-based high-throughput stability optimization of proteins for structural studies. Anal Biochem 357: 289–298). The apparent melting temperature is derived from the inflexion point of the curve and the value given represents the average of three separate experiments. All the curves show similar denaturation patterns. For cobalt, the fluorescence was quenched by the metal but interpretable curves were still obtained (off-set).
(0.74 MB TIF)

Figure S6 Manganese ion binding to LC180. Isothermal titration calorimetry measurements of manganese binding to (A) LC180 wild-type and (B) the D52A mutant. In each case, the upper plot shows the binding isotherm and the lower plot shoes the integrated values of each corresponding isotherm after subtracting the heat produced by the manganese dilution. For wild-type (A) the data were fitted with a model comprising two independent sites yielding Kds of 7.20 (±1.73) and 159.0 (±42.9) µM. Saturation of the interaction is not reached at a 1∶10 protein: ion molar ratio due to the low affinity of the second binding site. For the D52A mutant (B) the data were fitted with a model comprising a single site giving a Kd of 21.0 (±2.3) µM. The red points were not used for the curve fitting. The lack of the second binding site explains the lower molar ratio needed for saturation. Values for the thermodynamic parameters derived from the model fitting are shown in Supplementary Table S1.
(3.63 MB TIF)

Figure S7 LACV nucleocapsid (RNP) reconstitution system. Schematic of the procedure used to reconstitute recombinant LACV nucleocapsids *in vivo*. Cells were transfected with expression plasmids for the viral polymerase genes (N, L) and a minireplicon construct encoding a Renilla luciferase (Ren-Luc) gene flanked by viral promoter sequences (vRen). A co-transfected firefly luciferase (FF-Luc) serves as transfection control (not shown).
(6.74 MB TIF)

Figure S8 Cap-dependent transcription activity of wild-type and mutant LACV L-proteins. **A.** Immuno-staining of wild-type and mutant LACV L-protein expression. BSR-T7/5 cells grown on cover slips in 6-well dishes were transfected with 1 µg of T7-driven expression constructs for wt and mutants of LACV polymerase L, or were left untransfected (mock). At 24 h post-transfection, cells were fixed, permeabilised, and immune-stained using a rabbit polyclonal antiserum raised against recombinant LC180. **B and C.** Cap-dependent transcription of LACV reporter mRNA. Huh7 cells were transfected with IRES-containing LACV and FF-Luc plasmids as indicated, as well as with the minireplicon plasmid vREN. To destroy cap-dependent mRNA translation, an IRES-containing expression construct for polio virus 2APro was added to the plasmid mix. Empty vector (pTM1) and a proteolytically inactive mutant (2APro (G60R)) were used in parallel as controls. Cell lysates were assayed 24 h post transfection for REN-Luc (B) and FF-Luc activities (C). Luciferase counts were normalized to L activities with cotransfected pTM1. Mean values and standard deviations from three independent experiments are shown.
(6.86 MB TIF)

Table S1 Thermodynamic parameters for manganese binding to wild-type and D52A LC180 obtained from curve fitting to ITC data.
(0.01 MB DOCX)
