# Supplementary figures and images for: Bunyaviridae RNA Polymerases (L-Protein) Have an N-Terminal, Influenza-Like Endonuclease Domain, Essential for Viral Cap-Dependent Transcription
Source: PLoS Pathog. 2010 Sep 16;6(9):e1001101. doi: 10.1371/journal.ppat.1001101 (PMC2940753; doi:10.1371/journal.ppat.1001101)

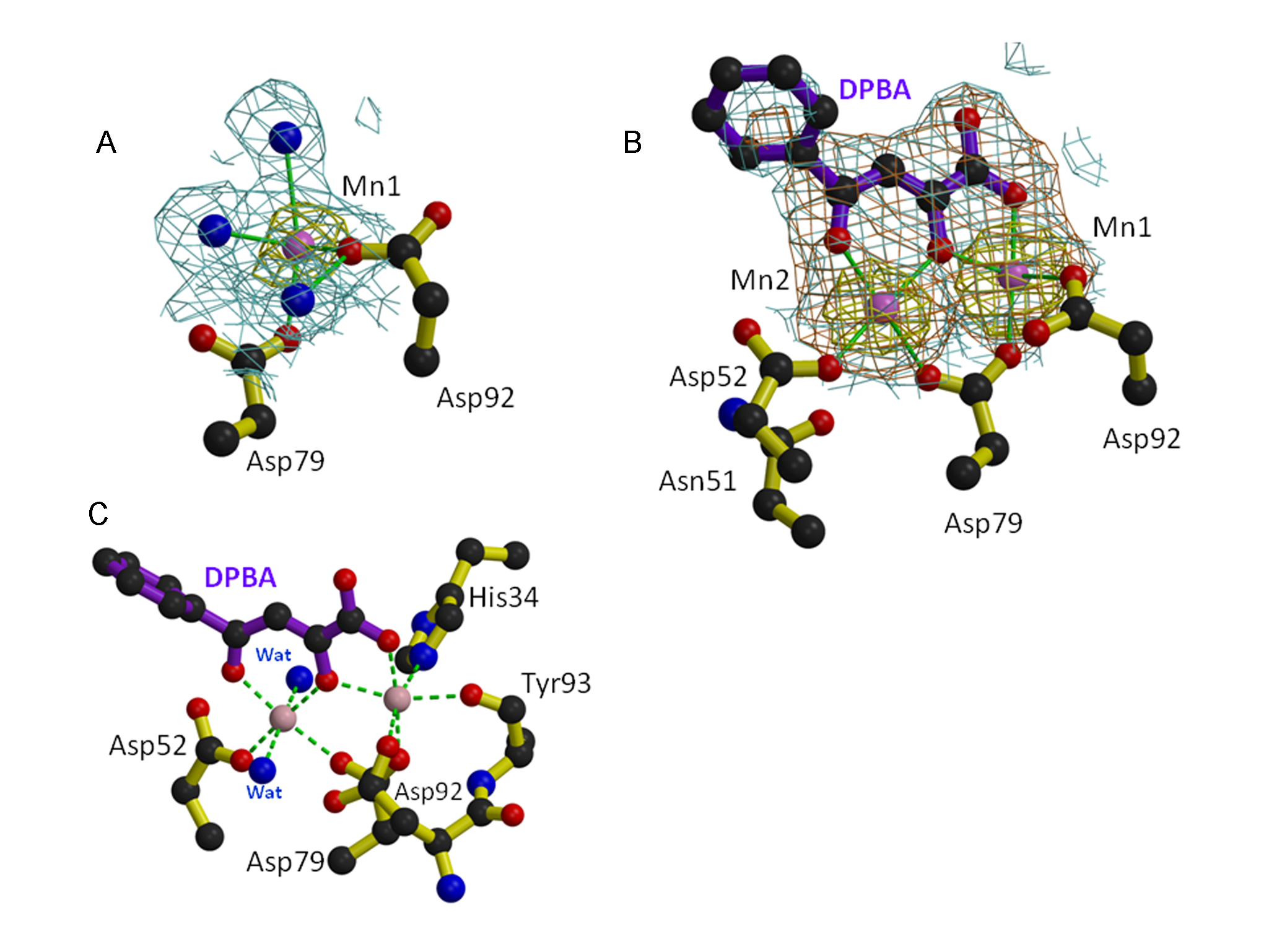

Supplement: Figure S1 — Metal ion and inhibitor binding in the active site of LC183. (a) Native LC183 structure with only one ion manganese bound. Blue: final 2fo-fc electron density at 0.95 σ (blue net). Anomalous difference map contoured at 4.0 σ (yellow net). Manganese ions and water molecules are represented by pink and blue spheres respectively. Mn1 has anomalous peak heights of between 7.3 and 11.5 σ for the four independent molecules in the asymmetric unit. (b) DPBA bound structure with two manganese ions. DPDA is in purple stick representation. Colours are as in (a) with in addition, the unbiased fo-fc positive difference electron density at 0.95 σ (brown net). Mn1 and Mn2 have anomalous peak heights of respectively between 17.8 and 20.9 σ and between 10.3 and 11.8 σ, for the four independent molecules in the asymmetric unit. (c) Diagram showing full cation and DPBA co-ordination (green dotted lines). (1.55 MB TIF) [file ppat.1001101.s001.tif]

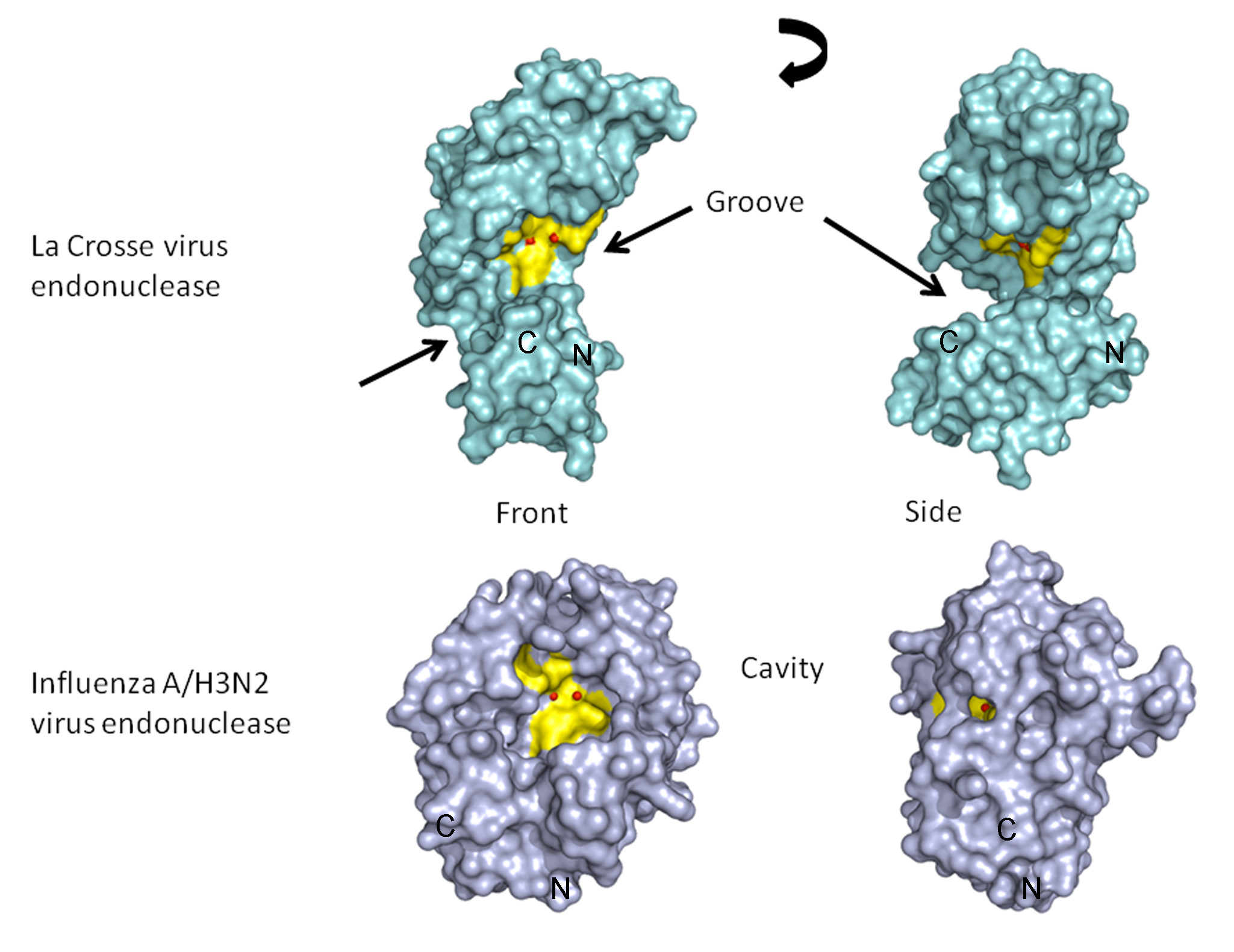

Supplement: Figure S2 — Active site accessibility of the LACV and Influenza virus endonuclease structures. Surface representation of the LACV (top, cyan) and Influenza (bottom, violet) endonuclease structures in two orientations after superposition. The conserved active site residues are coloured in yellow and the two metal ions are red spheres. The LC183 active site is in an open channel formed between two lobes of the protein into which double-stranded nucleic acid might fit. In contrast the PA-Nterm active site is less accessible in the bottom of a deep depression cavity with an estimated volume of 536 Å3 (programme POCASA). N- and C-termini of the proteins are marked. (1.93 MB TIF) [file ppat.1001101.s002.tif]

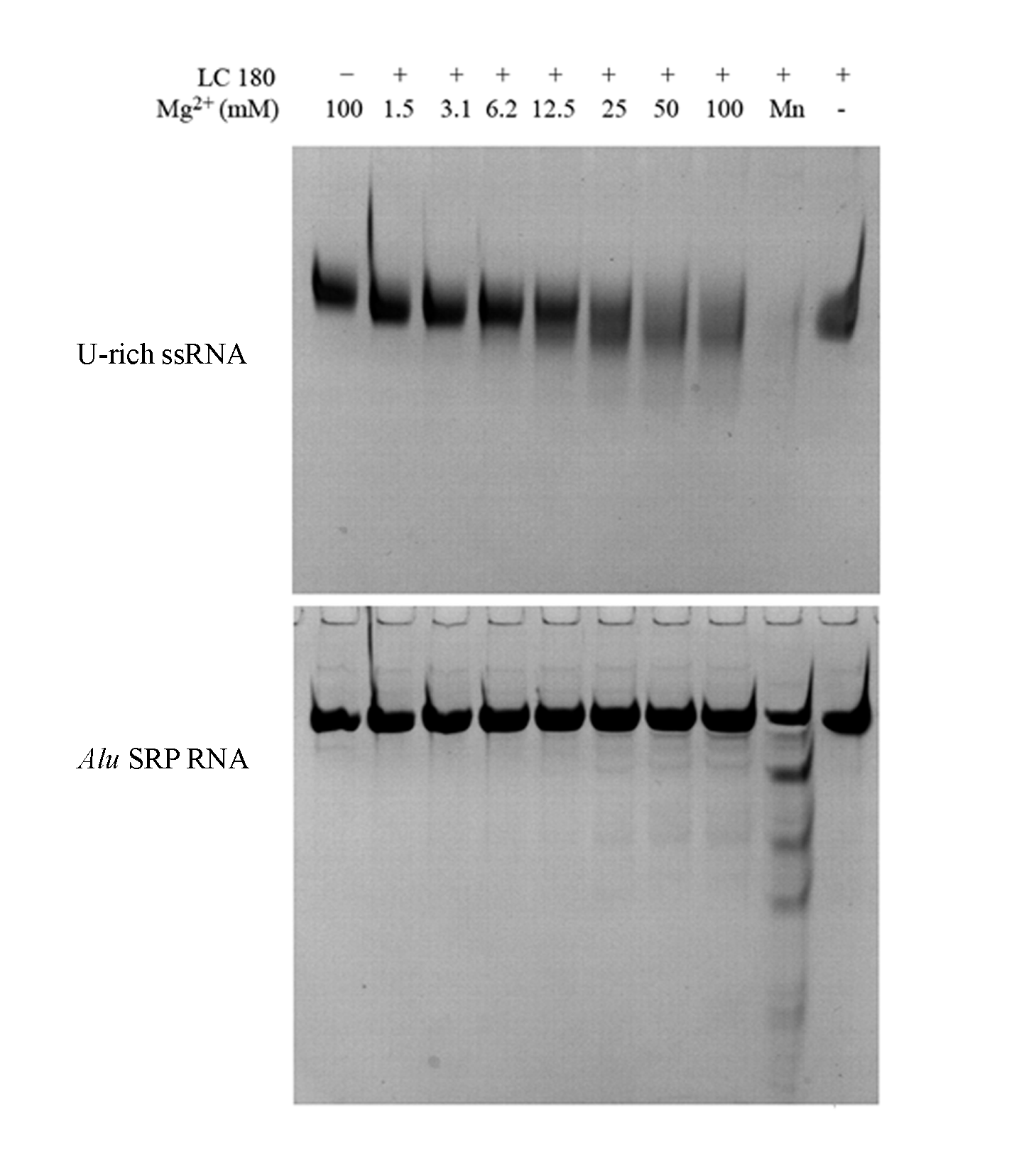

Supplement: Figure S3 — Nuclease assays of LC180 in the presence magnesium. Increasing concentrations of magnesium ions were tested in endonuclease activity assays with 6 µM of protein concentration and 7.5 or 6 µM concentration of U-rich or Alu SRP RNAs respectively. Significant RNA digestion is only observed above 12.5 mM concentration of magnesium. Even at the highest concentrations the activity with magnesium is far less than that with 2 mM manganese. (0.83 MB TIF) [file ppat.1001101.s003.tif]

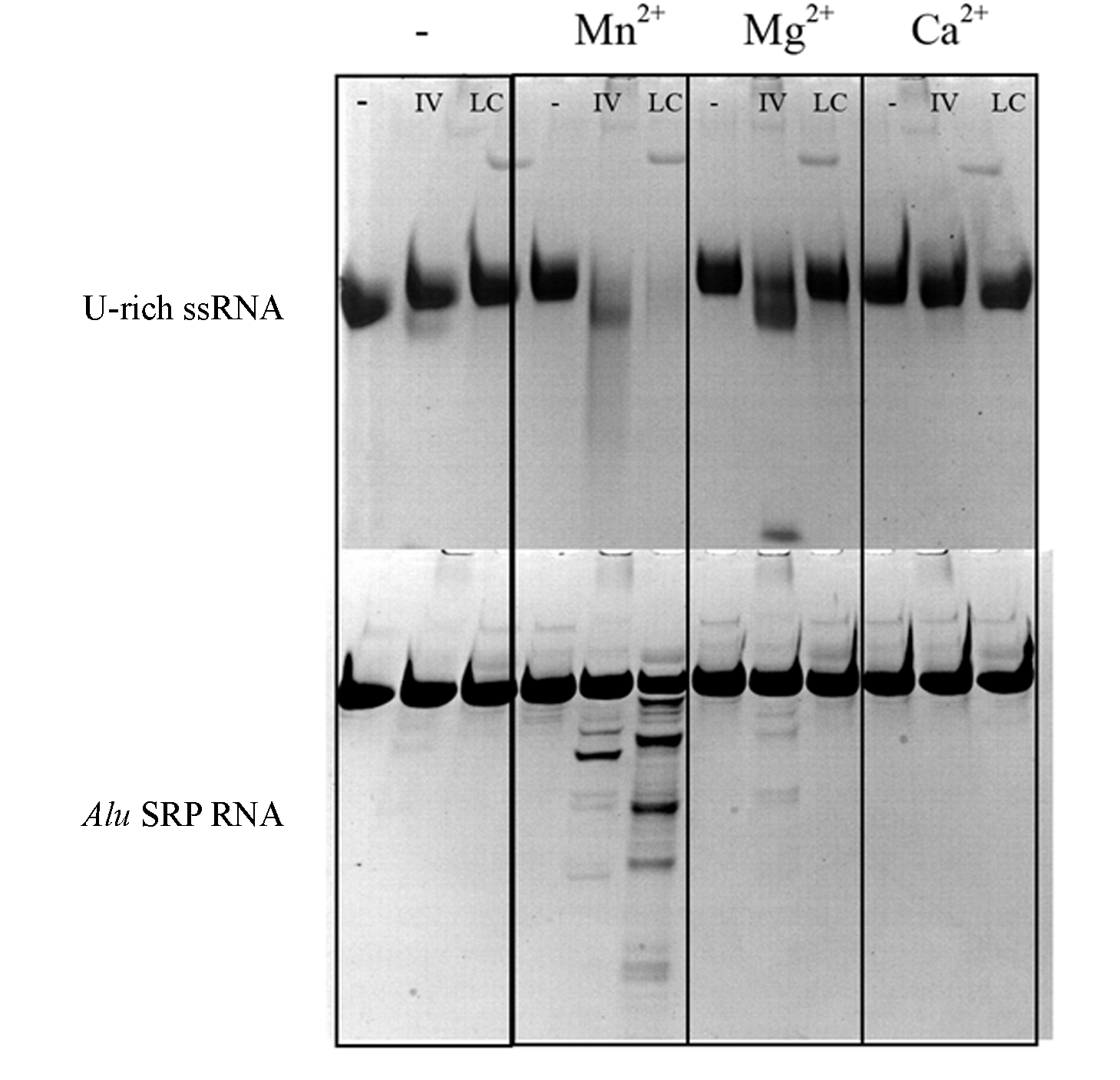

Supplement: Figure S4 — Divalent cation dependent nuclease activity of LC180 (LC) in comparison with the influenza endonuclease PA-Nterm (IV). Reactions were carried out with 10 µM concentration of protein and 12.6 µM or 10 µM concentration of U-rich and Alu SRP RNAs respectively for 50 min at 37°C in the presence of 2 mM manganese, magnesium or calcium. The maximum activity is achieved with manganese ions for both proteins, although the influenza endonuclease has some activity in the presence of magnesium ions, whereas LC180 does not. Neither have activity in the presence of calcium ions. The LACV protein has higher activity with the highly structured Alu SRP RNA than the influenza endonuclease. (4.79 MB TIF) [file ppat.1001101.s004.tif]

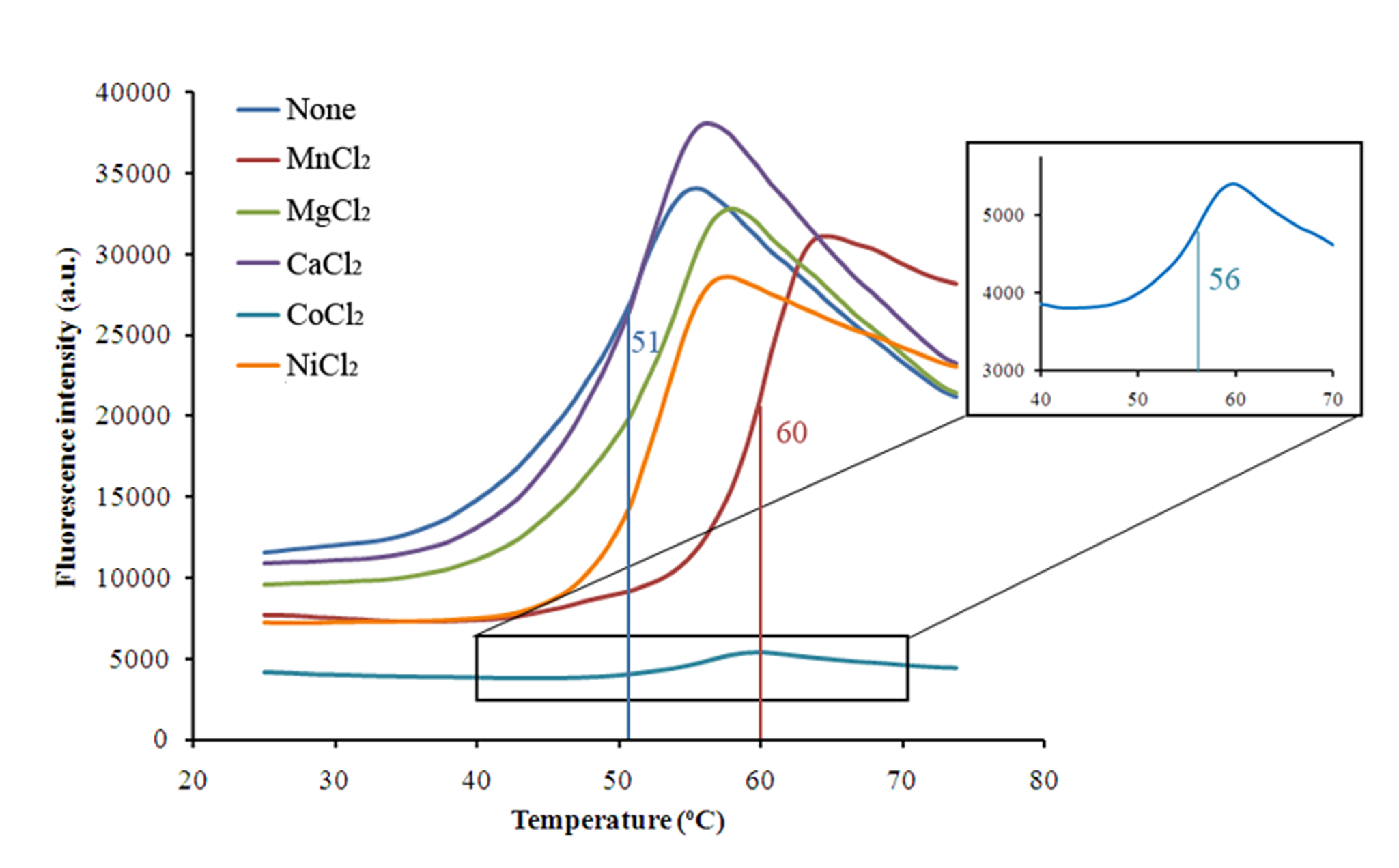

Supplement: Figure S5 — Single Thermofluor experiment of LC180 in the presence of 2 mM of various divalent cations. Thermofluor experiments were performed as described in the methods (Ericsson UB, Hallberg BM, Detitta GT, Dekker N, Nordlund P (2006) Thermofluor-based high-throughput stability optimization of proteins for structural studies. Anal Biochem 357: 289–298). The apparent melting temperature is derived from the inflexion point of the curve and the value given represents the average of three separate experiments. All the curves show similar denaturation patterns. For cobalt, the fluorescence was quenched by the metal but interpretable curves were still obtained (off-set). (0.74 MB TIF) [file ppat.1001101.s005.tif]

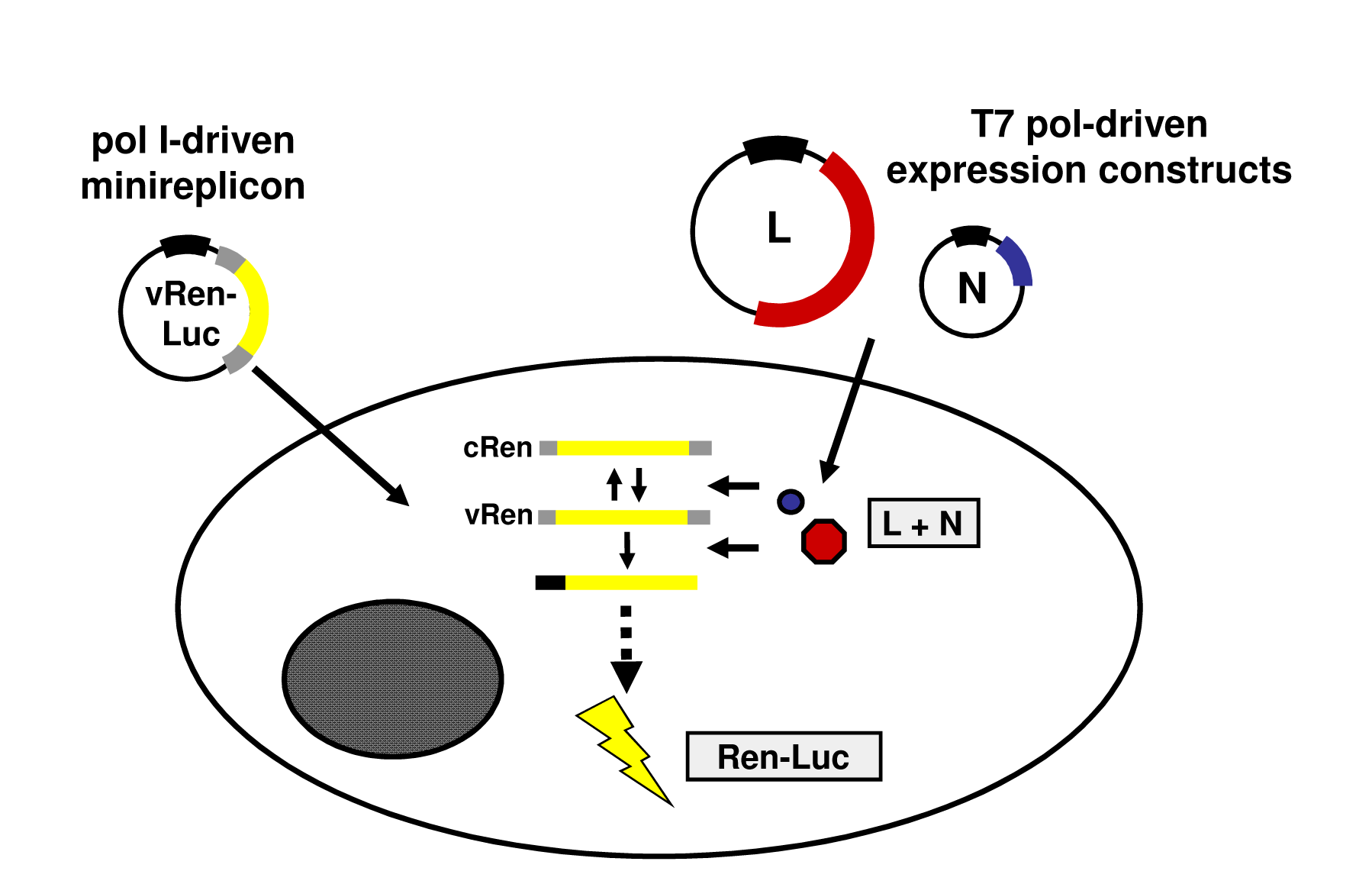

Supplement: Figure S7 — LACV nucleocapsid (RNP) reconstitution system. Schematic of the procedure used to reconstitute recombinant LACV nucleocapsids in vivo. Cells were transfected with expression plasmids for the viral polymerase genes (N, L) and a minireplicon construct encoding a Renilla luciferase (Ren-Luc) gene flanked by viral promoter sequences (vRen). A co-transfected firefly luciferase (FF-Luc) serves as transfection control (not shown). (6.74 MB TIF) [file ppat.1001101.s007.tif]

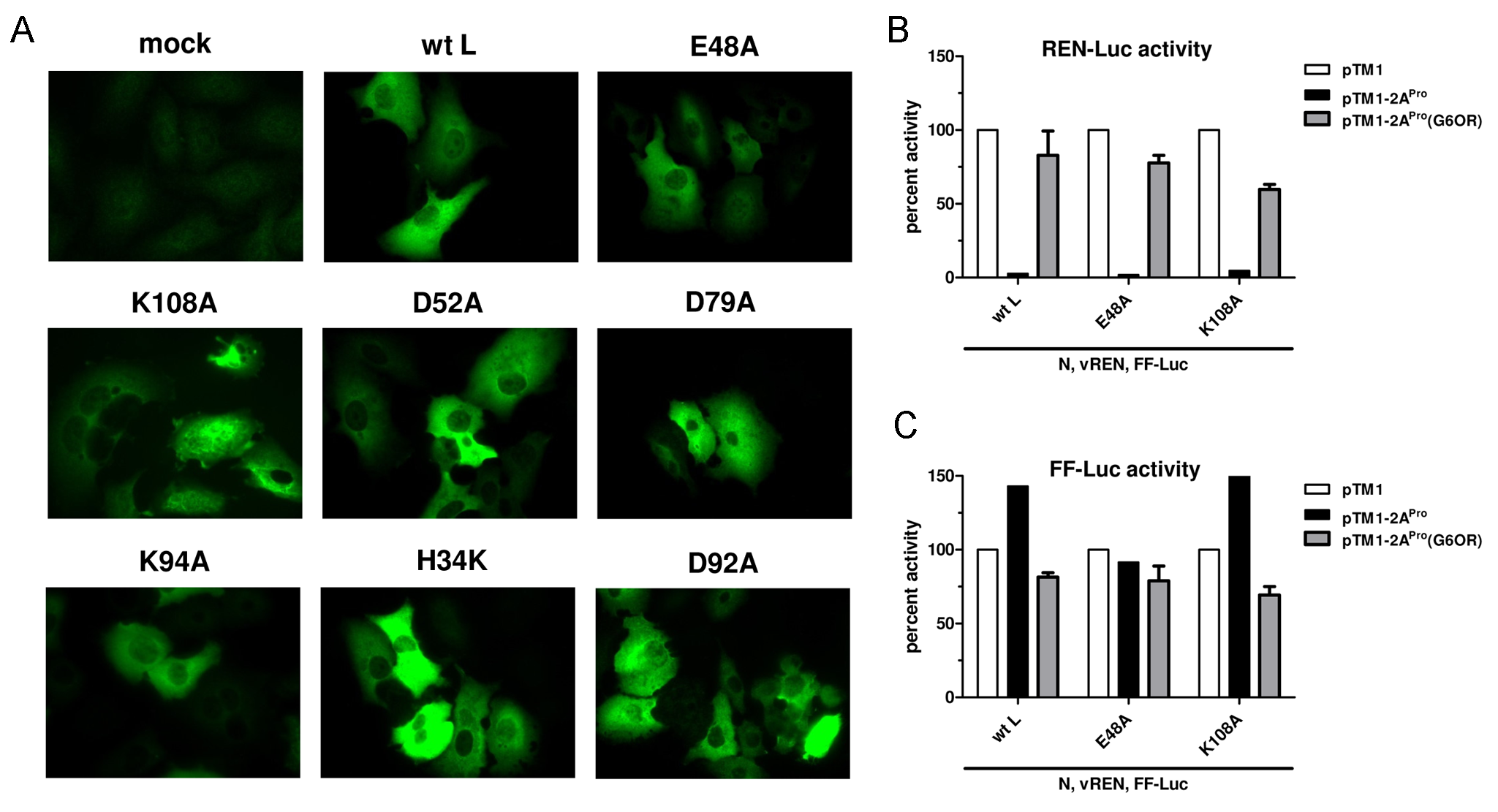

Supplement: Figure S8 — Cap-dependent transcription activity of wild-type and mutant LACV L-proteins. A. Immuno-staining of wild-type and mutant LACV L-protein expression. BSR-T7/5 cells grown on cover slips in 6-well dishes were transfected with 1 µg of T7-driven expression constructs for wt and mutants of LACV polymerase L, or were left untransfected (mock). At 24 h post-transfection, cells were fixed, permeabilised, and immune-stained using a rabbit polyclonal antiserum raised against recombinant LC180. B and C. Cap-dependent transcription of LACV reporter mRNA. Huh7 cells were transfected with IRES-containing LACV and FF-Luc plasmids as indicated, as well as with the minireplicon plasmid vREN. To destroy cap-dependent mRNA translation, an IRES-containing expression construct for polio virus 2APro was added to the plasmid mix. Empty vector (pTM1) and a proteolytically inactive mutant (2APro (G60R)) were used in parallel as controls. Cell lysates were assayed 24 h post transfection for REN-Luc (B) and FF-Luc activities (C). Luciferase counts were normalized to L activities with cotransfected pTM1. Mean values and standard deviations from three independent experiments are shown. (6.86 MB TIF) [file ppat.1001101.s008.tif]
