# Supplementary material for: Bunyaviridae RNA Polymerases (L-Protein) Have an N-Terminal, Influenza-Like Endonuclease Domain, Essential for Viral Cap-Dependent Transcription
Source: PLoS Pathog. 2010 Sep 16;6(9):e1001101. doi: 10.1371/journal.ppat.1001101 (PMC2940753; doi:10.1371/journal.ppat.1001101)
Supplement: Table S1 — Thermodynamic parameters for manganese binding to wild-type and D52A LC180 obtained from curve fitting to ITC data. (0.01 MB DOCX) [file ppat.1001101.s009.docx]

|  | **D52A** |  | **WT** |  |
| --- | --- | --- | --- | --- |
|  |  |  |  |  |
| N1 (sites) | 0.925 | +/- 0.027 | 0.857 | +/- 0.129 |
| K1 (M-1) | 4.80E+04 | +/- 3.24E+03 | 1.45E+05 | +/- 4.97E+04 |
| ΔH1 (cal/mol) | -3485 | +/- 132.12 | -2591 | +/- 109.6 |
| ΔS1 (cal/mol/deg) | 9.725 |  | 14.87 |  |
| N2 (sites) |  |  | 1.028 | +/- 0.301 |
| K2 (M-1) |  |  | 6.57E+03 | +/- 4.47E+02 |
| ΔH2 (cal/mol) | |  | -8.67E+03 | +/- 3.46E+03 |
| ΔS2 (cal/mol/deg) | |  | -11.63 |  |
